# NFκB inhibitors as a potential novel hypothesized treatment for psoriasis

**DOI:** 10.1590/S1516-31802011000600011

**Published:** 2011-12-01

**Authors:** Nima Derakhshan

**Affiliations:** I MD. Research Associate, Shiraz Nephro-Urology Research Center, Shiraz University of Medical Sciences, Shiraz, Iran.

Psoriasis is a chronic, relapsing autoimmune skin disease that affects an estimated 1-3% of the world's population.^1^ Identification of novel target pathways is necessary for treatment of this clinical enigma. Genetic, immunohistochemical and animal and human pharmacological studies support a possible role for nuclear factor kappa B (NFκB) activation in the pathogenesis of psoriasis.

Among the genes considered to be important in psoriasis are the ones involved in apoptosis and cytokine production, via pathways such as those mediated by NFκB, which is a transcription factor that regulates hundreds of genes, including many involved in apoptosis.^2,3^ Thus, it may be considered to be a link between cell-mediated and keratinocyte-mediated mechanisms, which are believed to be the two different pathogenetic mechanisms of psoriasis.^4^

Immunohistochemical analysis on skin biopsies has revealed overexpression of NFκB in psoriatic epidermis, in comparison with normal epidermis.^5^ The Rel/NFκB transcription factors play a central role in numerous cellular processes, including keratinocyte proliferation and differentiation. Lizzul et al. designed a study in which a phosphorylation-specific antibody was used, and evaluated their results by means of immunohistochemistry. They proved that there was no expression of active NFκB in the normal epidermis of non-psoriatic skin, whereas a basal level of constitutive active phosphorylated NFκB/RelA was present in uninvolved epidermis in psoriasis patients. There was also significant upregulation of active phosphorylated NFκB/RelA in the epidermis from psoriatic plaques.^6^ These data suggest that activation of NFκB plays a significant role in the pathogenesis of psoriasis.

Several pharmacological studies have revealed that certain antipsoriatic drugs exert their action through inhibition of NFκB activation pathways. Acitretin has been found to decrease the expression of signal transducer and activator of transcription 1 (STAT1) and NFκB in nuclei of the human keratinocyte cell line (HaCaT), as determined by means of immunocytochemistry and Western blot. This suggests that the antipsoriatic effects of acitretin may be related to inhibition of nuclear translocations of STAT1 and NFκB.^7^ It has been shown that dimethylfumarate (DMF) inhibits NFκB translocation, which leads to its three possible antipsoriatic effects: (i) inhibition of pro-inflammatory cytokine production and adhesion molecule expression; (ii) inhibition of dendritic cell differentiation; and, at higher concentrations, (iii) induction of apoptosis.^8^ Rottlerin, which has been attributed with antipsoriatic properties, is not only an antioxidant but also a potent NFκB inhibitor.^9^ One potential mechanism of action for tumor necrosis factor-targeting agents in psoriasis is downregulation of NFκB transcriptional activity. Etanercept therapy in patients with psoriatic arthritis has been found to result in a significant decrease in NFκB, through inhibition of NFκB pathway.^10^

Animal studies have also suggested a possible role for NFκB signaling pathways in the pathogenesis of psoriasis.^11,12^ Marchetti et al. conducted a study that proved that increased expression of NFκB is an essential step in inducing a psoriasis-like inflammatory syndrome in mice.^13^ Functional blockade of NFκB by means of NFκB inhibitors in transgenic murine and human epidermis produced hyperplastic epithelium *in vivo.* Application of a pharmacological NFκB inhibitor to intact skin was found to result in epidermal hyperplasia. In contrast, overexpression of active p50 and p65 NFκB subunits in transgenic epithelium produced hypoplasia and growth inhibition.^14^

Studies on cytokine production pathways have also pointed towards a key role for NFκB activation in the pathogenesis of psoriasis. Transforming growth factor-alpha has been found to induce interleukin-6 in the human keratinocyte cell line (HaCaT) by means of transcriptional activation, possibly through NFκB activation.^15^ Insulin-like growth factor II (IGF-II), which has been shown to be higher in psoriatic patients, has been found to increase the interleukin-6 levels, possibly through NFκB activation.^16^

Emerging data gathered from several genetic, immunohistochemical and animal and human pharmacological studies support a possible role for NFκB activation pathways in the pathogenesis of psoriasis. This suggests that NFκB inhibitors may prove to be invaluable in treating psoriasis. The fact that this pathway is not activated in normal keratinocytes and only targets psoriatic skin lesions makes NFκB inhibitors a potential novel addition to the antipsoriatic weaponry.
